# Effects of short-term fasting on spontaneous activity and excess post-exercise oxygen consumption in four juvenile fish species with different foraging strategies

**DOI:** 10.1242/bio.051755

**Published:** 2020-09-29

**Authors:** Xiuming Li, Yaoguang Zhang, Shijian Fu

**Affiliations:** 1 Laboratory of Evolutionary Physiology and Behavior, Chongqing Key Laboratory of Animal Biology, Chongqing Normal University, Chongqing, 400047, China; 2 Key Laboratory of Freshwater Fish Reproduction and Development (Education Ministry), Key Laboratory of Aquatic Science of Chongqing, School of Life Sciences, Southwest University, Chongqing, 400715, China

**Keywords:** Food deprivation, Spontaneous behavior, Excess post-exercise oxygen consumption (EPOC), Fishes

## Abstract

To investigate the effects of short-term fasting on spontaneous activity and excess post-exercise oxygen consumption (EPOC) in sit-and-wait carnivorous southern catfish (*Silurus meridionalis*), active carnivorous black carp (*Mylopharyngodon piceus*), active herbivorous grass carp (*Ctenopharyngodon idellus*) and active filter-feeding silver carp (*Hypophthalmichthys molitrix*), each species was divided into a control group and a fasting group (deprived of food for 14 days). Both groups were maintained at 25°C and, at the end of the experimental period, the total movement distance (TMD), percent time spent moving (PTM), ventilation frequency (*V*_f_), pre-exercise oxygen consumption (M(•)O_2_) and EPOC response of the experimental fish were measured. The TMD and PTM obtained for the control group of southern catfish were lower than those found for the control groups of the three active species. Short-term fasting resulted in decreases in the TMD and PTM of the southern catfish and black carp and increases in the TMD of grass carp and silver carp. The *V*_f_ of southern catfish was significantly higher than those of grass carp and silver carp, whereas the latter was also significantly higher than that of black carp. Short-term fasting resulted in significant increases in the *V*_f_ and decreases in the pre-exercise M(•)O_2_ of southern catfish and silver carp. Southern catfish and black carp exhibited lower peak post-exercise M(•)O_2_ and recovery rates, and relatively higher EPOC magnitudes than grass carp and silver carp. Short-term fasting exerted no significant effects on the peak post-exercise M(•)O_2_, but resulted in relatively higher EPOC magnitudes in the four fish species. These results suggest that (1) different fish species exhibit significantly different levels of spontaneous activity and post-exercise M(•)O_2_ profiles with different characteristics and that (2) short-term fasting exerts different effects on the level of spontaneous activity in four fish species with different foraging strategies.

## Abstract (precis)

**Summary:** This study compares the spontaneous behavior and exhaustive exercise of four warm-water fish.

## INTRODUCTION

Due to the spatial and temporal heterogeneity of food resource distributions, wild animals often face the stress of food scarcity and even starvation (Wang et al., 2006; McCue, 2010; Yeakel et al., 2018; Ezzat et al., 2019). Fasting exerts significant effects on the morphologies, physiological functions and behavioral patterns of fish, and these effects vary depending on the fish’s size, fasting duration and species (Wang et al., 2006; McCue, 2010; Fu et al., 2012; Zaldúa and Naya, 2014). In addition, the early life stages of fish species might be more affected by changes in food resources (Bar, 2014), and fish species with different foraging habits have different morphological, physiological and behavioral characteristics that might exhibit different acclimation strategies to short-term fluctuations in food resources due to unknowns in future food availability (Fu et al., 2007, 2009a; Caruso et al., 2011). Thus, studies on the impact of short-term fasting on fish might be important because the fish are frequently exposed to this stress in a culture environment or in nature and the effects of this stress varies among fish species with different foraging strategies (Hevrøy et al., 2011; López-Luna et al., 2013; Morshedi et al., 2017).

Spontaneous activity is an intuitive manifestation of the physiological status and behavioral characteristics of fish and is used for daily routine tasks, such as foraging activity and safeguarding territories (Hubbs and Blaxter, 1986; Xia et al., 2014). The spontaneous activity of fish can be assessed by certain indexes, such as the total movement distance (TMD) and percent time spent moving (PTM) (Fu et al., 2009a; Xia et al., 2014, 2015). The initial responses of many fish species to fasting involve increases in spontaneous activity to increase the chance of finding more food (Pulgar et al., 1999; Miyazaki et al., 2000; Killen et al., 2011). However, the increased spontaneous activity under fasting conditions will inevitably increase the energy consumption in the fish. Therefore, the strategy would be to lower spontaneous activity, which is indicative of energy conserving behavior, due to a compromise between food acquisition and energy conservation (Fu et al., 2012). The behavior patterns of fasting fish might depend on the fasting duration and the foraging habits of fish. Several studies have found that fish species tend to increase their locomotive activity under conditions of food scarcity and reduce their locomotive and metabolic activities as much as possible after fasting for longer than a specific time limit (Wieser, 1991; Sogard and Olla, 1996). Because fish with different foraging strategies likely have different expectations for the probability of obtaining food again, the behavioral strategies of fish in the face of fasting stress might be different (Croy and Hughes, 1991; Pulgar et al., 1999). Thus, characterizing the changes in behavioral characteristics of fish with different foraging strategies will help researchers evaluate the health and nutritional status of fish under short-term fasting. The first aim of this study was to test whether short-term fasting exerts different effects on the levels of spontaneous activity in fish species with distinct foraging strategies.

Exhaustive exercise plays an important role in the lives of fish species because it is closely related to some daily activities, such as capturing food, escaping from predators and swimming through rapids (Kieffer, 2000). Fish often need to perform exhaustive exercise in fishways with a high velocity of water. The increased oxygen consumption (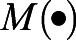
O_2_) above resting levels after exhaustive exercise, which is termed ‘excess post-exercise oxygen consumption’ (EPOC), reflects the increased oxygen consumption required to restore the tissue and cellular stores of oxygen and high-energy phosphates, the biochemical imbalances in metabolites, such as lactate removal and glycogen replenishment, and other functions such as ionic and osmotic balance (Gaesser and Brooks, 1984; Gleeson and Hancock, 2002; Lee et al., 2003a). It has been suggested that the maximum 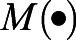
O_2_ can be elicited by manual chasing to exhaustion, and the peak post-exercise 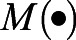
O_2_, immediately after exercise, can be used as an indicator of the aerobic capacity of fish species (Fu et al., 2009b; Tang et al., 2010). The EPOC magnitude is closely related to the anaerobic capacity of fish species, and the recovery rate of post-exercise metabolism serves as an index of their ability to recover from exhaustive exercise (Fu et al., 2007, 2009b; Zeng et al., 2010). Previous studies have shown that fasting influences a number of physiological (e.g. energy reserves) and biochemical (e.g. enzyme levels) indexes in fish (Sheridan and Mommsen, 1991; Tang et al., 2010) that could potentially affect swimming metabolism. Fasting may have an effect on the ability of fish to endure exhaustive exercise, which may affect their survival success swimming through waterways. However, relatively few studies have investigated the effects of fasting on the EPOC of fish species (Tang et al., 2010; Luo et al., 2013). Both the peak post-exercise 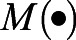
O_2_ and the EPOC magnitude of Nile tilapia (*Oreochromis niloticus*) exhibit significant decreases with increases in the fasting duration (Luo et al., 2013). However, short-term fasting (2 weeks) results in a significant increase in the peak post-exercise 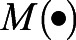
O_2_ and EPOC magnitude of Chinese catfish (*Silurus asotus*) (Tang et al., 2010). Therefore, the second aim of this study was to investigate whether short-term fasting exerts different effects on the EPOC of fish species with distinct foraging strategies.

To address our research objectives, we selected four fish species with different foraging strategies (Ding, 1994): southern catfish (*Silurus meridionalis*), which is a sit-and-wait carnivorous fish; black carp (*Mylopharyngodon piceus*), which is an active carnivorous fish; grass carp (*Ctenopharyngodon idellus*), which is an active herbivorous fish; and silver carp (*Hypophthalmichthys molitrix*), which is an active filter-feeding fish. These are four important cultured species within their respective foraging modes and are some of the most abundant fish species in the middle and upper reaches of the Yangtze River in China (Duan et al., 2002). We assessed the two indexes of spontaneous activity (TMD and PTM), the ventilation frequency (*V*_f_), the pre-exercise 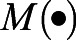
O_2_ and the EPOC responses of the four fish species with different foraging strategies before and after short-term fasting.

## RESULTS

### Body mass and body length

**Fig. 1.**
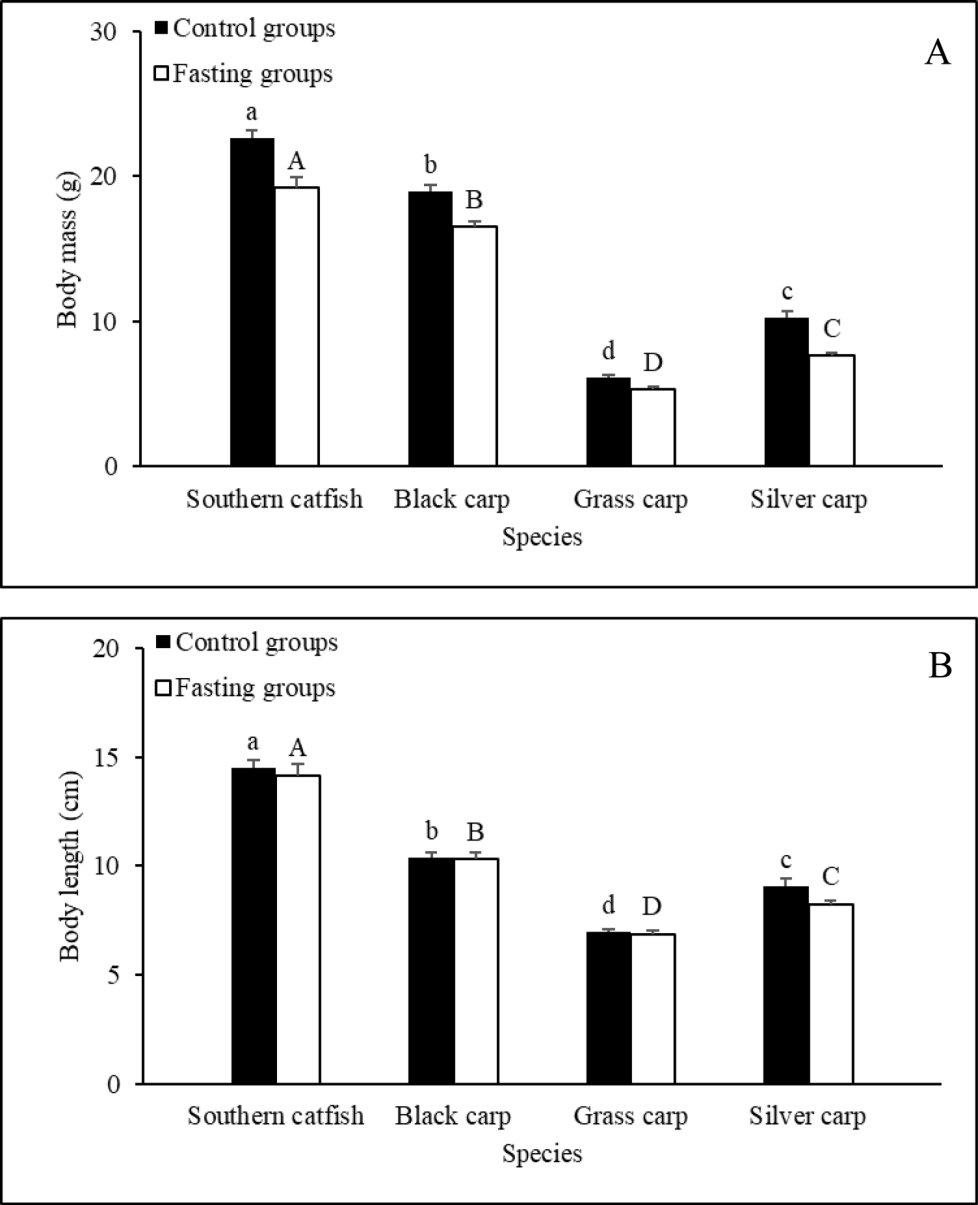
**Effects of fasting on the body mass and body length of the experimental fishes (*N*=10, mean±s.e.).** The values of the control groups without a common lowercase letter (a,b,c,d) are significantly different. The values of the fasting groups without a common capital letter (A,B,C,D) are significantly different (*P*<0.05).

Significant differences in the body masses and body lengths were found among the control groups (or fasting groups) of the different fish species (*P*<0.001) (Table 1; Fig. 1A and B). Among all fish species, short-term fasting had significant effects on body masses (*P*<0.001), whereas it produced no significant effects on the body lengths (Table 1; Fig. 1A and B).

**Table 1.**
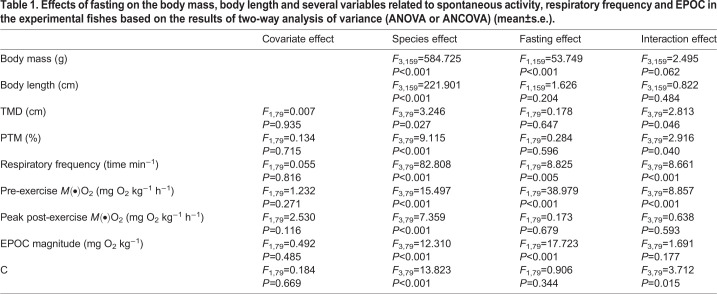
**Effects of fasting on the body mass, body length and several variables related to spontaneous activity, respiratory frequency and EPOC in the experimental fishes based on the results of two-way analysis of variance (ANOVA or ANCOVA) (mean±s.e.).**

### Spontaneous motion

**Fig. 2.**
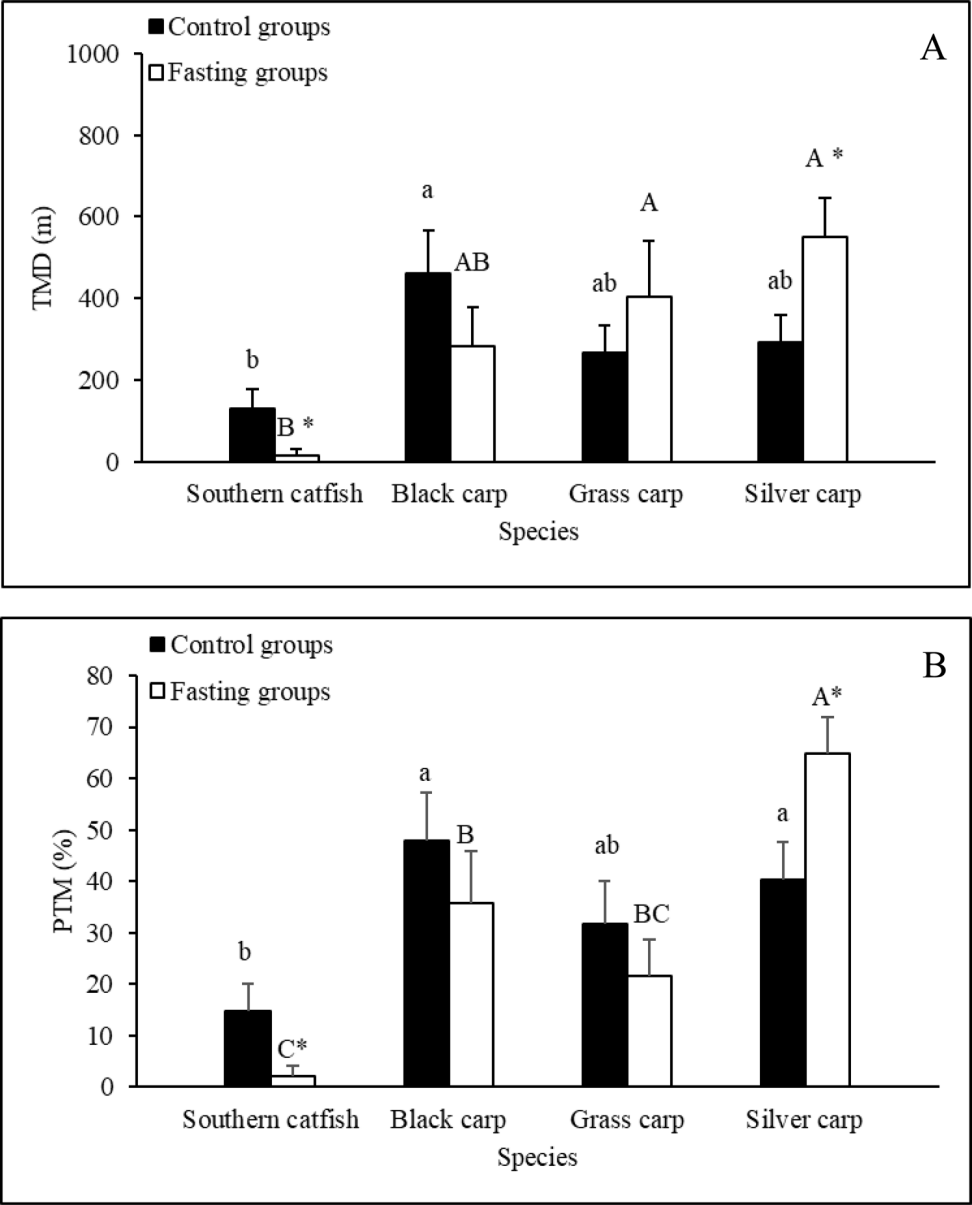
**Effects of fasting on spontaneous activity in the experimental fishes (*N*=10, mean±s.e.).** TMD (m), total movement distance; PTM (%), percent time spent moving. The values of the control groups without a common lowercase letter (a,b,c,d) are significantly different. The values of the fasting groups without a common capital letter (A,B,C,D) are significantly different. An asterisk (*) denotes a significant difference between the control and fasting groups of the same fish species (*P*<0.05).

Among the control groups, southern catfish exhibited the lowest TMD value, which was significantly lower than that of black carp (Fig. 2A), and the PTM of southern catfish was significantly lower than those of black carp and silver carp (Fig. 2B). Short-term fasting led to significant 88% and 87% decreases in the TMD and PTM of southern catfish, respectively, but 88% and 61% increases in the TMD and PTM of silver carp, respectively (Fig. 2A and B).

### *V*_f_

**Fig. 3.**
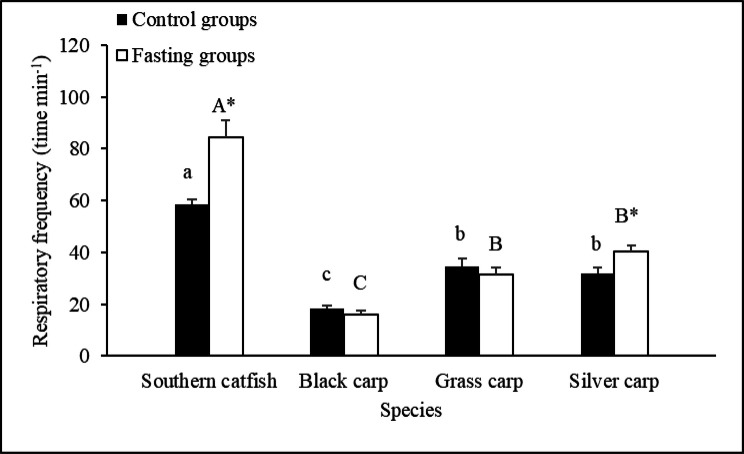
**Effects of fasting on the respiratory frequencies of the experimental fishes (*N*=10, mean±s.e.).** The values of the control groups without a common lowercase letter (a,b,c,d) are significantly different. The values of the fasting groups without a common capital letter (A,B,C,D) are significantly different. An asterisk (*) denotes a significant difference between the control and fasting groups of the same fish species (*P*<0.05).

Among the control groups, the *V*_f_ of southern catfish was significantly higher than those of grass carp and silver carp, whereas both grass carp and silver carp have significantly higher *V*_f_ than black carp (Fig. 3). Short-term fasting resulted in 44% and 26% increases in the *V*_f_ of southern catfish and silver carp, respectively but had no significant effect on the *V*_f_ of black carp and grass carp (Fig. 3).

### EPOC

#### Pre-exercise 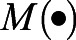
O_2_

**Fig. 4.**
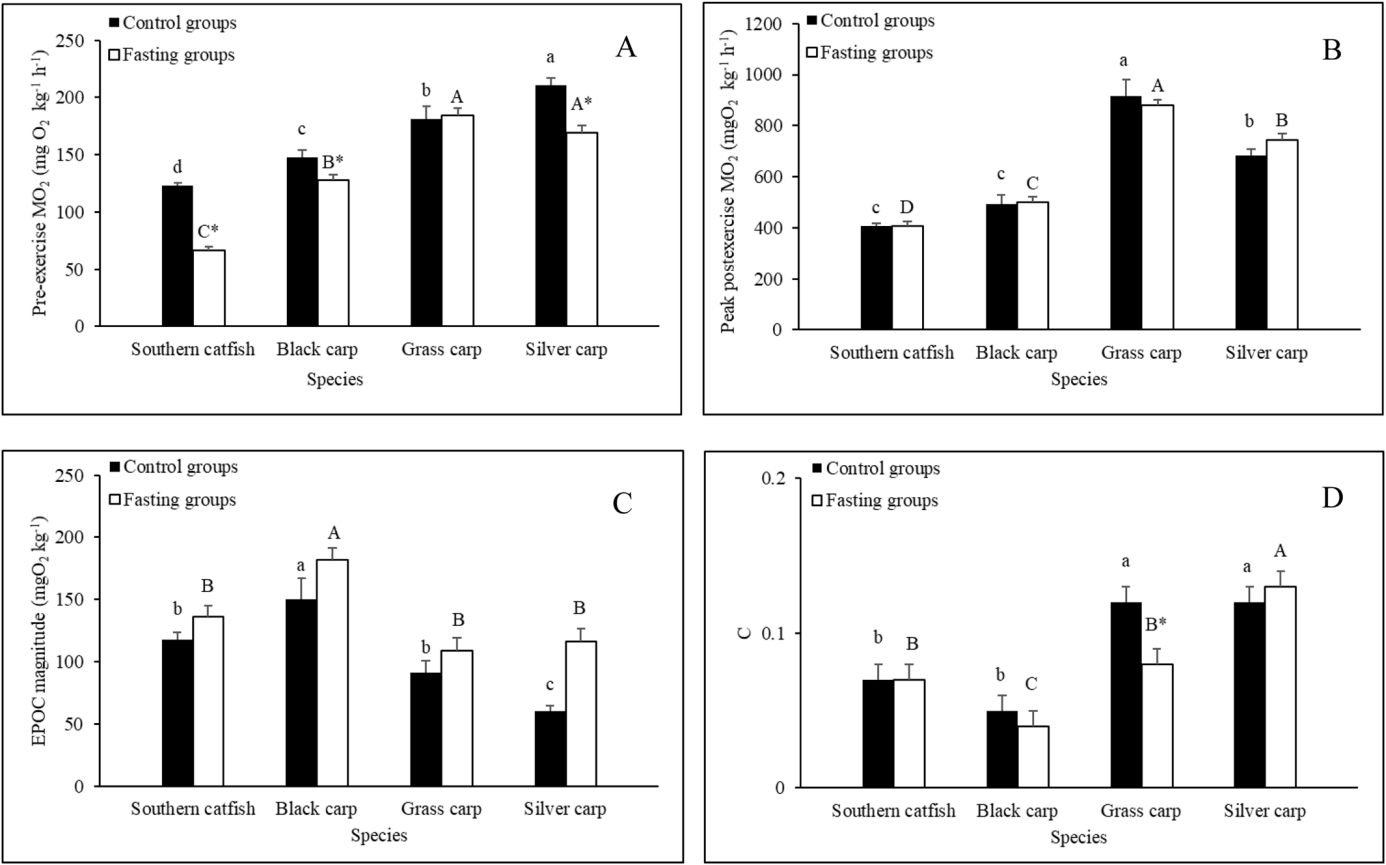
**Effects of fasting on the excess post-exercise oxygen consumption (EPOC) responses of several juvenile fishes (*N*=10, mean±s.e.).** The values of the control groups without a common lowercase letter (a,b,c,d) are significantly different. The values of the fasting groups without a common capital letter (A,B,C,D) are significantly different. An asterisk (*) denotes a significant difference between the control and fasting groups of the same fish species (*P*<0.05). The c value is the rate of recovery from exhaustive exercise.

Among the control groups, the pre-exercise 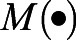
O_2_ levels of southern catfish and black carp were significantly lower than those of grass carp and silver carp (Fig. 4A). Short-term fasting resulted in 48%, 14% and 21% decreases in the pre-exercise 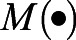
O_2_ values of southern catfish, black carp and silver carp, respectively, but had no significant effect on that of grass carp (Fig. 4A).

#### Peak post-exercise 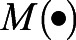
O_2_

**Fig. 5.**
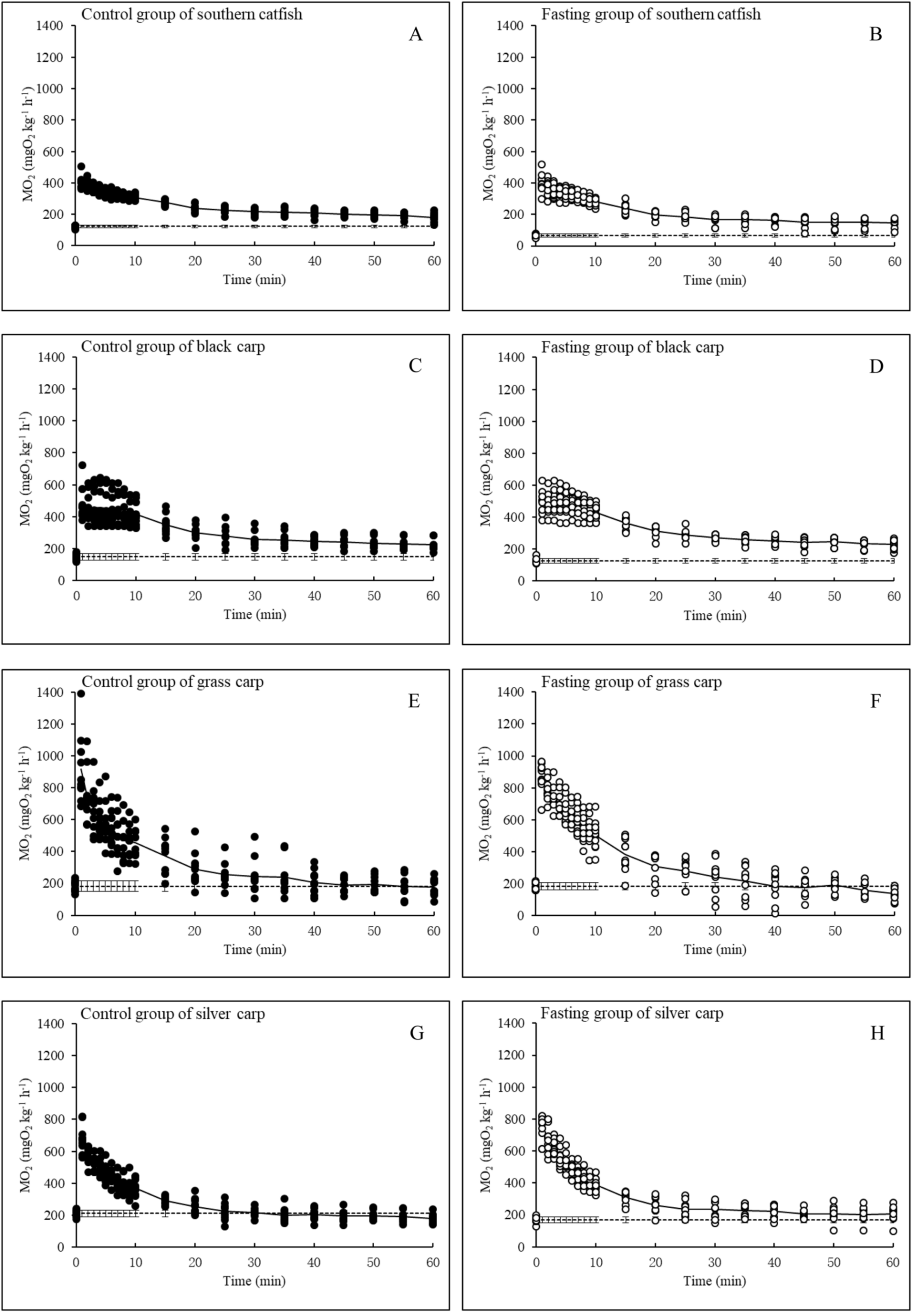
**Pre- and post-exercise 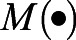
O_2_ levels of the control and fasting fish (*N*=10, mean±s.e.).**

The 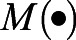
O_2_ increased immediately after exhaustive exercise and then returned to the pre-exercise level (Fig. 5). Among the control groups, the peak post-exercise 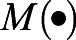
O_2_ value of grass carp was significantly higher than that of silver carp, which was significantly higher than those of southern catfish and black carp (Fig. 4B). Short-term fasting exerted no significant effect on the peak post-exercise 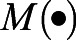
O_2_ values of the four fish species (Table 1; Fig. 4B).

### EPOC magnitude

Among the control groups, the EPOC magnitudes of southern catfish and grass carp were significantly lower than that of black carp and significantly higher than that of sliver carp (Fig. 4C). Short-term fasting exerted significant effects on the EPOC magnitudes of the four fish species (*P*<0.001) (Table 1; Fig. 4C).

### Recovery rate

Among the control groups, the values of c for grass carp and silver carp were significantly higher than those for southern catfish and black carp (Fig. 4D). Short-term fasting resulted in a significant 33% decrease in the value of c for grass carp but exerted no significant effect on the values of c for the other three species (Fig. 4D).

## DISCUSSION

### Effects of foraging mode and short-term fasting on spontaneous activity

According to animals adopting varying sets of coordinated behavioral tactics when searching for and attacking prey, animals are classified as ‘sit-and-wait’ or ‘widely foraging’ predators (Huey and Pianka, 1981; Tunney and Steingrímsson, 2012). Among the four non-fasting fish species evaluated in this study, sit-and-wait carnivorous southern catfish exhibited the lowest spontaneous activity level, as demonstrated by the finding that this species exhibited the lowest TMD and PTM values. This result, which was also found in a previous study (Fu et al., 2009a), was likely obtained because this species is at the top of the food chain in its habitat and tends to hide motionless most of the time. In contrast, two fish species with active habits, herbivorous grass carp and filter-feeding silver carp, showed relatively higher levels of spontaneous activity than southern catfish, which was expected and is consistent with the results of previous studies (Fu et al., 2009a,b; Nie and Fu, 2017). However, the TMD and PTM of carnivorous black carp were significantly higher than those of southern catfish and did not differ from those of grass carp and silver carp. This result might have been obtained because black carp feed on small prey rather than large prey, such as snails and aquatic insect larvae, in the field (Zhou et al., 2017) and adopt an active foraging strategy. These results suggest that the TMDs and PTMs of the four species exhibit distinct interspecific differences, as expected, and these differences might be associated with the different foraging modes of the species.

A change in behavior while experiencing food deprivation conditions have been reported for larvae, juveniles, and adults of some fish species (Laurence, 1972; Croy and Hughes, 1991; Pulgar et al., 1999; Miyazaki et al., 2000; Benhaïm et al., 2012), but to the best of our knowledge, there have been no studies comparing the differences in spontaneous activity among fish species with different foraging strategies under fasting conditions. In this study, short-term fasting led to 88% and 87% decreases in the TMD and PTM, respectively, of southern catfish (Fig. 2A and B). Although the effects were not significant, fasting decreased the TMD and PTM of black carp by 32% and 17%, respectively, compared with those of the non-fasting individuals. These results indicated that the two carnivorous species decreased their level of spontaneous activity, which might be conducive to further reductions in the energy expenditure during short-term fasting. This may be because the food resources of carnivorous fish are not readily available (the prey can escape the predator) and the variation in food resources is relatively large. In these species it may be more beneficial to reduce energy expenditure until food becomes abundant again. In contrast, short-term fasting resulted in 88% and 61% increases in the TMD and PTM, respectively, of silver carp. Although the difference was not significant, the TMD of fasting grass carp was 50% higher than that of the non-fasting individuals. This increased level of spontaneous activity in the fasting individuals was likely due to the small meal size of the two fish species and the relatively homogeneous distribution of their food resources (Novakowski et al., 2008). More frequent foraging activities would be beneficial for increasing the chance of encountering more food under fasting conditions, which might be the result of long-term acclimation in response to changes in food resources. An increase in spontaneous activity due to fasting is consistent with the results of previous studies in various fish species, such as an intertidal fish (*Girella laevifrons*) (Pulgar et al., 1999), Japanese flounder (*Paralichthys olivaceus*) (Miyazaki et al., 2000) and three-spined sticklebacks (*Gasterosteus aculeatus*) (Giles, 1987). Furthermore, although the difference in the TMD between the three cyprinid species was small, the PTM of silver carp was significantly higher than those of black carp and grass carp after short-term fasting. This finding might have been obtained due to the large distribution of plankton eaten by silver carp, and hence, fasting individuals spend more time on foraging.

### Effects of foraging mode and short-term fasting on the *V*_f_

The *V*_f_, which is a behavioral indicator that is often used to evaluate respiratory function in fish species, is affected by various environmental factors (Mattias et al., 1998; Rios et al., 2002; Fu et al., 2007; Zeng et al., 2012). Because the *V*_f_ is related to oxygen utilization by animals, we predicted that the fish species with active foraging strategies would have a higher *V*_f_ than those with inactive habits. However, in this study, the maximal *V*_f_ was observed in southern catfish (a sit-and-wait carnivorous fish) among the non-fasting fish species. A higher *V*_f_ (74–80 times min^−1^) of the species was found in a previous study (Fu et al., 2007). This result indicated that the *V*_f_ is not necessarily related to the foraging strategy. Southern catfish belong to Siluriformes, and the other three fish species belong to Cypriniformes. The gill structure of fish species with different taxonomic statuses is significantly different (Wilson and Laurent, 2002), and the relationship between the gill structure and the *V*_f_ of different fish species needs further study.

In this study, the *V*_f_ of southern catfish and silver carp increased after 14 days of fasting. The results for southern catfish was consistent with those obtained in a previous study, which found that short-term and prolonged fasting resulted in an increase in *V*_f_ (but an inevitable decrease after prolonged fasting) (Zeng et al., 2012). However, the same fasting cycle had no significant effect on the *V*_f_ of black carp and grass carp. A constant *V*_f_ was also previously found in traíras (*Hoplias malabaricus*) subjected to 240 days of fasting (Rios et al., 2002). The above-described data suggest that different fish species exhibit various respiratory responses to fasting. It is worth noting that an increase in 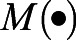
O_2_ is always accompanied by an increase in the *V*_f_ during some physiological activities, such as exercise and digestion (Fu et al., 2007). Thus, an elevated or constant *V*_f_ for fish species is probably necessary for extracting the necessary oxygen from and excreting the waste to the water environment during fasting. However, the present study found that fasting southern catfish and silver carp had lower pre-exercise 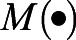
O_2_ levels and a higher *V*_f_ than non-fasting individuals, whereas fasting black carp had lower pre-exercise 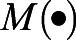
O_2_ levels and a constant *V*_f_ than non-fasting individuals. A similar unparallel change between the 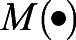
O_2_ levels and the *V*_f_ was also found in southern catfish during 32 days of fasting (Zeng et al., 2012). This might be because the ventilation volume and oxygen uptake rate in fish species are related not only to the *V*_f_ but also to the amplitude of the opercular beat (Rogers and Weatherley, 1983). Therefore, it is possible that southern catfish and silver carp increased their *V*_f_ but reduced the amplitudes of their opercular movement in this study. Another sit-and-wait species belonging to Siluriformes (Chinese catfish, *Silurus asotus*) also has a relatively high *V*_f_ (54–58 times min^−1^) (Li et al., 2012). The different response patterns of southern catfish might be due to its phylogeny (only species belonging to Siluriformes) and its extraordinarily high basal *V*_f_ under non-fasting conditions. The change might result in a higher respiratory efficiency and hence a conservation of the energy expenditure under food deprivation conditions (Mehner and Wieser, 1994; Fu et al., 2005b). Similarly, whether the change in the ventilation pattern of silver carp is due to its distinct filter foraging mode, which caused the feeding and opercular movements to co-evolve, also needs further investigation. Nevertheless, the present study revealed the following interesting phenomenon: the respiration mode changes after fasting, and the pattern of this change varies among species.

### Effects of foraging mode and short-term fasting on EPOC

In this study, the peak postexercise 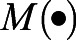
O_2_ levels and recovery rate (i.e. ‘c’) of herbivorous grass carp and filter-feeding silver carp were relatively higher, whereas the EPOC magnitudes were relatively lower, than those of carnivorous southern catfish and black carp. The higher peak 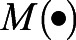
O_2_ levels and the higher c values of herbivorous and filter-feeding fishes suggests that these species have a higher aerobic capacity and exhibit faster recovery after exhaustive exercise than carnivorous species, which might be beneficial for their active lifestyle and hence more pursuits of physiological activities, such as frequent foraging, migration, and predator avoidance. These observations are consistent with previous studies that found that carnivorous southern catfish and black carp exhibit a lower aerobic swimming performance (critical swimming speed) than most other fishes in the same habitat, such as common carp (*Cyprinus carpio*), Chinese bream (*Parabramis pekinensis*) and qingbo (*Spinibarbus sinensis*) (Fu et al., 2009a; Nie and Fu, 2017). This result may be because southern catfish use a strategy of ambush predation, whereas black carp mainly prey on some benthic animals with lesser degree of motility, such as mollusks, clams, snails, shrimp and aquatic insect larvae, in nature (Ding, 1994; Zhou et al., 2017). In contrast, the larger EPOC magnitudes and hence higher anaerobic capacities of the carnivorous southern catfish and black carp than grass carp and silver carp might be beneficial for their ambush hunting activities or chasing prey (Fu et al., 2009b). Herbivorous grass carp and filter-feeding silver carp must swim frequently all day to feed on both aquatic plants and algae or phytoplankton and zooplankton, and thus, these two species might not need a high anaerobic capacity due to their high aerobic capacity. In addition, the EPOC magnitude depends on the levels of anaerobic fuels (e.g. glycogen) (Luo et al., 2013). The glycogen stores and the ability to utilize these stores during exercise can potentially impact the EPOC profile (Zhang et al., 2018). Therefore, the different diets provided to different experimental fish might have different impacts on the glycogen levels in muscle and would ultimately exert an influence on EPOC. Further study is needed to confirm these findings.

In this study, fasting for 2 weeks exerted no significant effects on the peak postexercise 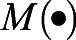
O_2_ of the four species. This result suggested that short-term fasting did not cause significant changes in the aerobic metabolic capacities of the four fish species with different foraging strategies. However, a previous study found that the peak post-exercise 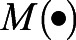
O_2_ of Nile tilapia significantly decreased after 2 weeks of fasting (Luo et al., 2013), whereas another study on Chinese catfish found that the same period of fasting resulted in a significant increase in the peak post-exercise 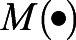
O_2_ (Tang et al., 2010). Thus, these results suggest that the effect of fasting on the peak 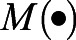
O_2_ level is species specific and might be related to the demand for power output available for physiological and behavioral activities during short-term fasting (Bennett, 1978; Luo et al., 2013).

In this study, short-term fasting resulted in relatively higher EPOC magnitudes in the four fish species (Fig. 4C). This result indicated that fasting individuals exhibits a relatively higher anaerobic metabolic capacity, which might be beneficial for important functions involving anaerobic metabolism, such as jumping out of the water, swimming through the rapids and escaping from predators. This phenomenon has also been found in fasting Chinese catfish (Tang et al., 2010). Furthermore, 2 weeks of fasting had no significant influence on the 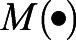
O_2_ recovery rates of southern catfish, black carp and silver carp, and this result was also observed in a recent study on Nile tilapia (Luo et al., 2013). However, the 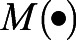
O_2_ recovery rate of grass carp decreased significantly after 2 weeks of fasting. This result might indicate a slower recovery from exhaustive exercise in this fish species after short-term fasting, which might have a negative impact on some physiological activities, such as escaping from predators.

In conclusion, the spontaneous activity level of nonactive carnivorous fish (southern catfish) was lower than those of three active species (black carp, grass carp and silver carp). After short-term fasting, the spontaneous activity levels of two carnivorous fish (southern catfish and black carp) decreased, whereas those of herbivorous grass carp and filter-feeding silver carp increased. The increase in the activity level of the two latter species might be an evolutionary adaptation of foraging mode to food resource fluctuations. The carnivorous fishes exhibited relatively lower aerobic capacities, higher anaerobic capacities and slower rates of recovery from exhaustive exercise compared with those of the herbivorous and filter-feeding fishes, possibly due to the foraging strategy of ambush predation or poor locomotion performance of mainly prey.

## MATERIALS AND METHODS

### Experimental animals

Juvenile experimental fish were purchased from local hatcheries (Hechuan or Beibei Chongqing, China) on the Jialing River. All experimental fish were 1-year-old juveniles and their sexes were not identified. Prior to the experiment, the fish were acclimated for 4 weeks in glass tanks (approximately 500 L). During this period, the temperature of dechlorinated freshwater was maintained at 25.0±0.5°C, and the oxygen content was kept above 7 mg l^−1^. The tank water was continuously filtered, and 10% of the water was replaced each day. The fish were fed to satiation twice daily at 09:00 and 18:00. All of the feed supplied to the fish was the same or similar to the feed used in aquaculture practice. Fillets of freshly killed loach (*Misgurnus anguillicaudatus*) were fed to the southern catfish, commercial floating pellets (Tongwei, Sichuan, China, dietary composition: 41.2% protein, 8.5% lipid, 25.7% carbohydrate and 12.3% ash) were fed to the black carp and grass carp, and a powder diet (Tongwei, Sichuan, China, dietary composition: 27.3% protein, 5.6% lipid, 33.1% carbohydrate and 8.2% ash) was fed to the silver carp. Any food and feces remaining after 3 h were removed. The photoperiod was maintained at a 12 h light:12 h dark cycle.

### Experimental protocol

After acclimation, 40 individuals (southern catfish: 22.63±0.55 g, 14.48±0.36 cm; black carp: 18.92±0.43 g, 10.36±0.24 cm; grass carp: 6.29±0.26 g, 6.88±0.12 cm; and silver carp: 10.26±0.46 g, 8.87±0.35 cm) of each species of fish were randomly divided into the control and fasting groups (20 fish per group). Although significant differences in size were found among the four fish species, which is mainly due to their different growth rates, all the fish were at the juvenile stage. Twenty fish from each group were placed in a separate cell composed of gray plastic boards (20 L) in an indoor circulating water system containing an ultraviolet lamp (11 W). According to the literature on postprandial metabolic responses, all the fish were fasted for a certain period (72 h for southern catfish and 24 h for black carp, grass carp and silver carp) (Fu et al., 2009a; Li et al., 2013, 2018). Ten fish from each control group were then used to determine the indexes of spontaneous activity and weighed to 0.01 g. The other ten fish from each control group were weighed and used to measure the pre-exercise 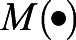
O_2_ and EPOC (see below). The fish in the fasting groups were deprived of food for 14 days; subsequently, ten fish from each fasting group were used to determine the indexes of spontaneous activity and weighed to 0.01 g, and the other ten fish from each fasting group were weighed and used to measure the pre-exercise 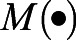
O_2_ and EPOC. Prior to being weighed, the fish were removed from the cells and slightly anesthetized (neutralized MS222, tricaine methane sulfonate, 50 mg l^−1^) in a 1-L container for 2–3 min until they no longer exhibited normal reflexes to tactile stimuli. The holding conditions (temperature, oxygen content and photoperiod) during the experimental period were consistent with those during the acclimation period.

### Measurements and calculations

#### Spontaneous activity

To assess the effects of fasting on the parameters of spontaneous activity in the experimental fishes, each individual fish was transferred to a white plastic bucket (60 L) and acclimated for 30 min. The diameter of the bucket was 40 cm and the water depth was maintained at 20 cm. The spontaneous activities of each fish were recorded for 20 min with video cameras (ST-399, Stjiatu Corporation, China) connected to a computer, and all the videos were analyzed with EthoVision XT 9 software (EthoVision XT 9, Nodus, Netherlands). In each video, the individual fish were detected by static subtraction with a sampling rate of 25 frames per second. The TMD (m) and PTM (%) of each fish over the course of 20 min were calculated to provide an indication of the spontaneous activity level of the fish (Fu et al., 2015; Nie and Fu, 2017).

#### *V*_f_ and pre-exercise 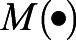
O_2_

To assess the effects of fasting on the *V*_f_ and pre-exercise 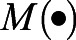
O_2_, the fish were individually placed in a continuous-flow respirometer chamber (see the structure described in Fu et al., 2005a) and allowed to acclimate for another 24 h. The 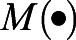
O_2_ level was measured three times at 2 h intervals over the course of 6 h, and the mean was defined as the pre-exercise 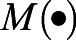
O_2_ value. The following formula was used to calculate the 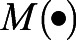
O_2_ level (mg O_2_ kg^−1^ h^−1^):(1)
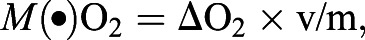
where ΔO_2_ is the difference (mg O_2_ L^−1^) in the oxygen concentration between the experimental chamber and the control chamber (chamber without fish), v is the water flow rate in the chamber (L h^−1^) and m is the body mass of the fish (kg).

The dissolved oxygen concentration was measured at the outlet of the chamber using an oximeter (HACH, HQ_30d_, USA). The flow rate of water through the respirometer chamber was measured by collecting the water outflow from each chamber. The flow rate in each chamber was adjusted to ensure 70% saturation of the dissolved oxygen concentration in the outlet water and thus avoid undue stress on physiological processes. Light was maintained throughout the experimental period to minimize the effect of circadian rhythms on 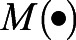
O_2_ (Fu et al., 2006). The *V*_f_ was determined using a stopwatch. Specifically, the number of opercular movements per minute was recorded three times at 2 h intervals over the course of 6 h, and the mean was defined as the *V*_f_ (Fu et al., 2007).

### EPOC

After measuring the pre-exercise 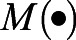
O_2_ levels of the experimental fishes (see above), an individual fish was gently removed from the respirometer chamber and exercised by being manually chased for 1–2 min to an exhausted state in a circular container (73 cm in diameter with a water velocity of approximately 65 cm s^−1^) (Fu et al., 2009b). The fish were considered exhausted when they were no longer responsive to manual chasing (Zeng et al., 2010). After being chased, the fish were immediately returned to the respirometer chamber. The measured flow rate was approximately 0.45 l min^–1^, and a 99% exchange of water was achieved within 1 min in a 0.08 L chamber (Steffensen, 1989). The 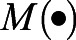
O_2_ values were measured 1, 2, 3, 4, 5, 6, 7, 8, 9, 10, 15, 20, 25, 30, 35, 40, 45, 50, 55 and 60 min after exercise. The 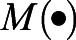
O_2_ levels were calculated using the formula above.

The following parameters were quantified to obtain a description of EPOC: (1) pre-exercise metabolic rate (mg O_2_ kg^−1^ h^−1^), oxygen consumption before exercise; (2) peak post-exercise metabolic rate (mg O_2_ kg^−1^ h^−1^), observed maximum oxygen consumption during the recovery process after exercise; and (3) EPOC magnitude (mg O_2_ kg^−1^), excess oxygen consumption above the pre-exercise 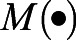
O_2_ during the recovery process.

The relationship between the post-exercise 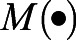
O_2_ (Y, mg O_2_ kg^−1^ h^−1^) and the time after exercise (X, min) is described by the following equation:(2)
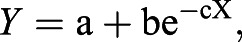
where a is the pre-exercise 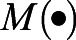
O_2_, b is the 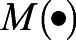
O_2_ increment, and c is the recovery rate. A high c value indicates a fast rate of recovery from exhaustive exercise (Pang et al., 2019).

This study complied with the current law of the country in which it was performed and was approved by the Animal Care and Use Committee of the Key Laboratory of Animal Biology of Chongqing (permit number Zhao-20,151,010-01). The study was performed in strict accordance with the recommendations in the Guide for the Care and Use of Animals at the Key Laboratory of Animal Biology of Chongqing, China.

### Data analysis

Statistical analyses were conducted with Excel software (Microsoft Corporation, 2003) and SPSS 17.0 software (IBM, 2008). The values are expressed as the mean±standard error (SE), and *P*<0.05 was used as the level of statistical significance. The effects of species and fasting on the body mass and body length were assessed by two-way analysis of variance (ANOVA). The effects of species and fasting on all variables of spontaneous activity and EPOC were assessed by two-way analysis of covariance (ANCOVA). The body mass was used as the covariate for all the variables except TMD and PTM, for which the body length was used as the covariate. ANOVA or ANCOVA was followed by a least significant difference multiple comparison test for TMD, PTM, *V*_f_, pre-exercise MO_2_ and recovery rate.
